# Roughness of surface decorated with randomly distributed pillars

**DOI:** 10.1038/s41598-018-34446-z

**Published:** 2018-10-30

**Authors:** Paweł Weroński

**Affiliations:** Jerzy Haber Institute of Catalysis and Surface Chemistry, Polish Academy of Sciences, Niezapominajek 8, PL-30239 Krakow, Poland

## Abstract

We have presented a quantitative analysis of roughness of planar surfaces decorated with randomly distributed, cylindrical pillars, disks, or cavities. We have described the roughness in terms of the surface power spectral density (PSD). First, we have derived a general equation for the PSD of such surfaces. Then, we have found the PSD for the special case of statistically isotropic, circular areas. We have demonstrated that the PSD provides quantitative information on the radius of the circular area, dimensions of the pillar, and surface coverage. We have also discussed the numerical method of extracting the parameters from experimental PSD data obtained from discrete Fourier transform of surface scanning measurements.

## Introduction

Surface roughness is an important factor determining a number of physico-chemical phenomena and processes. It plays an essential role in adhesion, contact mechanics, friction, surface wearing, reflection of electromagnetic waves, structural coloring, sealing, microfluidics, electrochemistry, and surface wettability^1,2^. Therefore, controlling surface roughness is a key problem in various fields of science and industry. It has to be managed to produce high-quality optics, anti-reflective and corrosion resistance coatings, superhydrophobic surfaces, microdevices, automotive parts, or manipulators, to mention just a few. There are also many fascinating features of living beings, which are conditioned upon the proper surface roughness. Let us just recall the unusual adhesion abilities of gecko feet^3^, self-cleaning properties of lotus leaves^4^, structural coloration common in animals and flowers^5^, or antireflective properties of moth eye^6^. These natural phenomena have been a great inspiration for scientists of various disciplines, who have produced high-tech surfaces with sophisticated functionalities. A variety of experimental methods have been exploited for the purpose. These are template-based techniques, micro- and nanolithography, plasma treatment, self-assembly and self-organization, chemical deposition, layer-by-layer deposition, colloidal assembly, phase separation, and electrospinning^4^. It is worth to mention that colloidal particle deposition is a simple method for controlling surface roughness, at the nanolevel, in a wide range^7^.

Once we have produced the surface, we may need to quantitatively determine its roughness. For that, investigators usually employ contact or optical profilometry^8,9^. Atomic force microscopy and white light interferometry or laser scanning confocal microscopy are typical examples of the former and latter. From the surface profile measurements, we can find a number of quantitative parameters and functions characterizing the surface roughness, such as the root mean square roughness and power spectral density (PSD), respectively. The latter provides us with a lot of valuable information because it describes the distribution of signal power over the spatial frequency. This function is also routinely used for theoretical investigations of surface roughness. To the best of our knowledge, however, no investigations of roughness of model, planar surfaces decorated with cylindrical pillars, disks, or cavities have been carried out so far. Therefore, the main aim of our research is to shed more light on the roughness of such surfaces. Specifically, we want to present a general, analytical description of the PSD of non-overlapping, monodisperse pillar layers, valid for various radial distribution functions. Based on this description, we have also demonstrated a new method of determination of important pillar layer parameters.

Obviously, there exist a number of experimental tools that would allow us to precisely determine various parameters of such a simple system without calculating its PSD. For example, we could derive the pillar dimensions and surface coverage directly from AFM measurements, using software for image analysis. However, this is not the case for more complicated systems involving, e.g., polydisperse pillars, cones, spheres, or objects lacking rotational symmetry. Therefore, this paper is intended to be a first step toward better understanding more complicated systems met in real-world problems.

The outline of the paper is as follows. First, we have derived a general equation for the PSD of the surfaces. Next, we have obtained the PSD for the special case of statistically isotropic, circular areas. We have demonstrated that the PSD provides information on the size of investigated area, pillar dimensions, and pillar surface coverage. Finally, we have discussed our results in the context of potential application of the PSD for the quantitative determination of the pillar layer parameters.

## Analytical Considerations

### Fourier transform of pillar decorated surface

**Figure 1:**
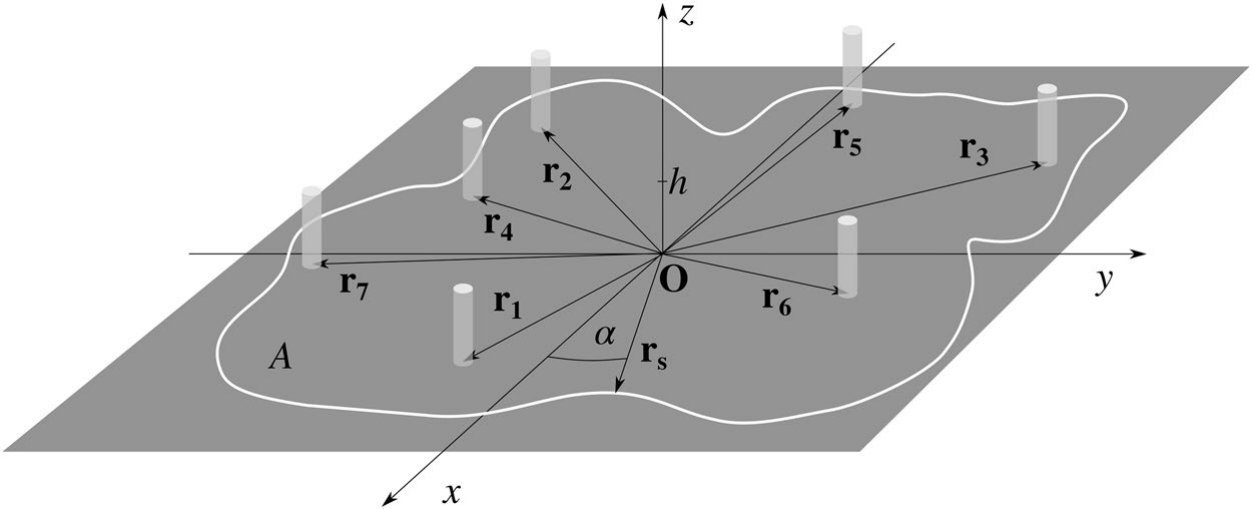
Scheme of planar area *A* in Cartesian coordinates, decorated with randomly distributed pillars. The area is enclosed by the curve **r**_s_(*α*)^18^.

Let us consider a planar area *A* in the Cartesian coordinate system shown in Fig. 1, enclosed by the curve **r**_**s**_(*α*), where *α* is the polar angle of the vector **r**_**s**_. The area is decorated with *N* identical, circular, randomly distributed pillars of the height *h* and is otherwise smooth. We can uniquely describe the system geometry by specifying the curve **r**_**s**_(*α*) and set of position vectors **r**_**j**_ = (*x*_*j*_, *y*_*j*_), *j* = 1, …, *N*, where *x*_*j*_ and *y*_*j*_ are the coordinates of the *j*-th pillar’s base center. Unless stated otherwise, for the sake of notation simplicity, we have normalized all dimensional parameters and variables by the pillar radius *a* to a power matching their physical dimensions, to make them dimensionless. In what follows, to neglect boundary effects, we have assumed that linear dimensions of the area *A* are many times larger than the pillar radius, i.e., that |**r**_**s**_(*α*)| ≫ 1 for any value of *α*.

Once we know the position vectors of all pillars, we can quantitatively describe the height of the pillar decorated surface. For that, we have used the function of height:

1 $$z({\bf{r}},{{\bf{r}}}_{{\bf{1}}},\ldots ,{{\bf{r}}}_{{\bf{N}}})={z}_{1}({\bf{r}},{{\bf{r}}}_{{\bf{1}}})+{z}_{2}({\bf{r}},{{\bf{r}}}_{{\bf{2}}})+\ldots +{z}_{N}({\bf{r}},{{\bf{r}}}_{{\bf{N}}})=\sum _{j=1}^{N}{z}_{j}({\bf{r}},{{\bf{r}}}_{{\bf{j}}}),$$

where

2 $${z}_{j}({\bf{r}},{{\bf{r}}}_{{\bf{j}}})={z}_{j}({\bf{r}}-{{\bf{r}}}_{{\bf{j}}})=\{\begin{array}{ccc}\,h & \,\Longleftrightarrow \, & |{\bf{r}}-{{\bf{r}}}_{{\bf{j}}}|\le 1\\ 0 & \,\Longleftrightarrow \, & |{\bf{r}}-{{\bf{r}}}_{{\bf{j}}}| > 1.\end{array}$$

**Figure 2:**
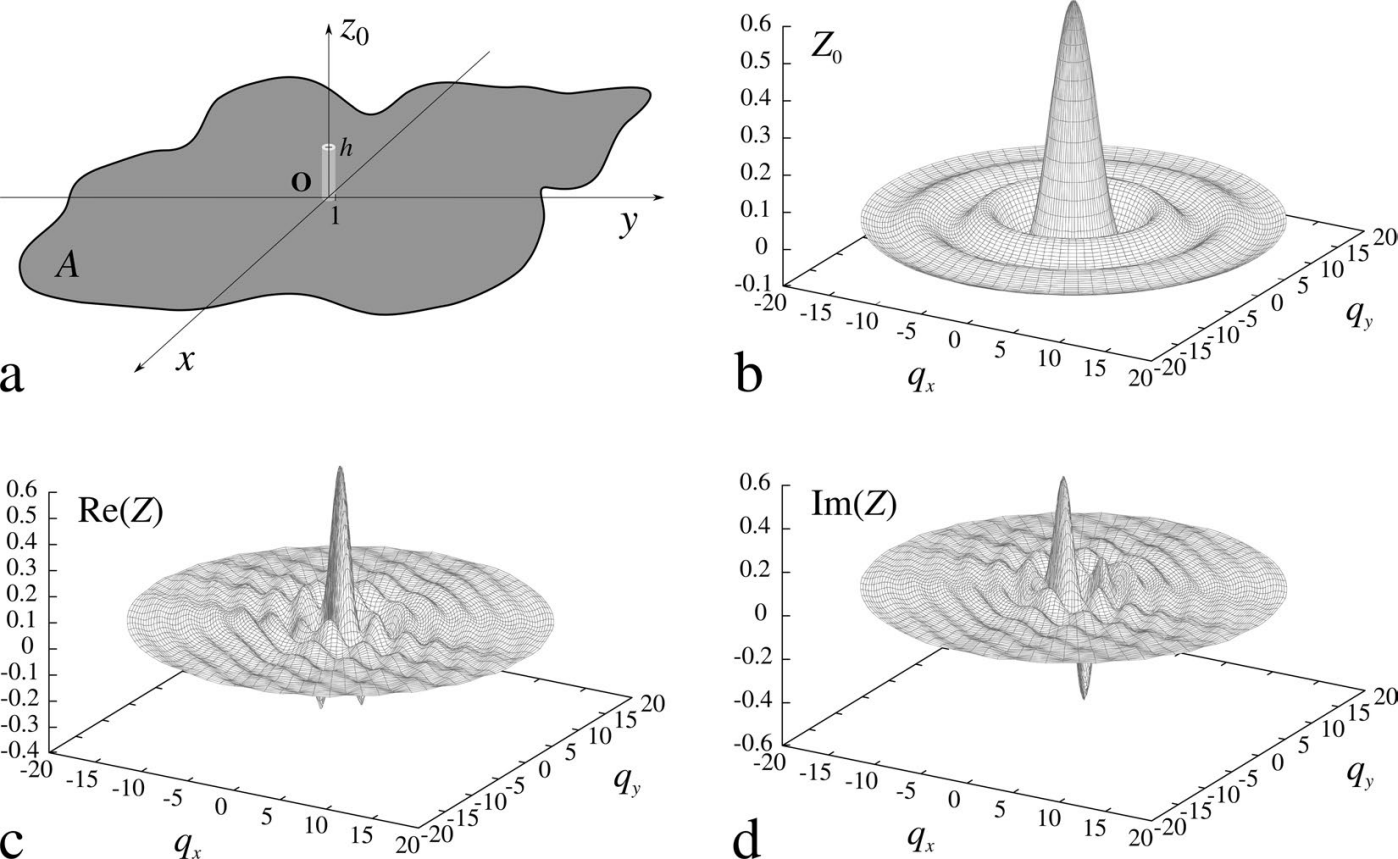
(**a**) Single pillar at the origin of Cartesian coordinates. (**b**) Fourier transform of the function of height shown in (**a**). Please note that the transform is a pure real function. Figures (**c**) and (**d**) present, respectively, the real and imaginary parts of Fourier transform calculated for the single pillar at the position (1, 1)^19^.

Equation (2) describes the function of height of the surface decorated with a single pillar located at **r**_**j**_. Mathematically, the single pillar system can be generated by translation of the pillar, originally located at the origin, by the vector **r**_**j**_. In Fig. 2a, we have presented the pillar at its original position. The function of height for this system has the canonical form

3 $${z}_{0}(r)=\{\begin{array}{ccc}\,h & \,\Longleftrightarrow \, & r\le 1\\ 0 & \,\Longleftrightarrow \, & r > 1.\end{array}$$

Please note that this function, because of axial symmetry of the system, depends on the modulus *r* of the vector **r** only.

The Fourier transform of *z*(**r**, **r**_**1**_, …, **r**_**N**_) over the finite area *A* equals

4 $$Z({\bf{q}},{{\bf{r}}}_{{\bf{1}}},\ldots ,{{\bf{r}}}_{{\bf{N}}})=F\{z({\bf{r}},{{\bf{r}}}_{{\bf{1}}},\ldots ,{{\bf{r}}}_{{\bf{N}}})\}=\sum _{j=1}^{N}F\{{z}_{j}({\bf{r}},{{\bf{r}}}_{{\bf{j}}})\}=\sum _{j=1}^{N}{Z}_{j}({\bf{q}},{{\bf{r}}}_{{\bf{j}}}),$$

where *Z*_*j*_(**q**, **r**_**j**_) is the Fourier transform of *z*_*j*_(**r**, **r**_**j**_) over *A* and **q** is the wave vector of the modulus *q* = 2*π*/*λ*. Here, *λ* denotes the dimensionless wavelength.

We can exploit the Fourier transform translation theorem to rewrite the single pillar transform as

5 $${Z}_{j}({\bf{q}},{{\bf{r}}}_{{\bf{j}}})={Z}_{0}(q)\exp (\,-\,i{{\bf{r}}}_{{\bf{j}}}\cdot {\bf{q}}),$$

where *Z*_0_(*q*) is the Fourier transform of *z*_0_(*r*) over *A* and *i* is the imaginary unit. Please note that the function *Z*_0_(*q*) depends on the wavenumber *q* only. It is easy to show that the transform equals

6 $${Z}_{0}(q)=\frac{1}{{(2\pi )}^{2}}{\int }_{A}{z}_{0}(r)\exp (\,-\,i{\bf{r}}\cdot {\bf{q}}){\rm{d}}{\bf{r}}=\frac{h}{2\pi }\frac{{J}_{1}(q)}{q},$$

where *J*_1_(*q*) is the first order Bessel function of the first kind. In Eq. (6), as well as in the whole paper, we have assumed that *q* > 0. Thus, in the very special case of pillar at the origin, its Fourier transform is a purely real function. In Fig. 2b, we have presented the function *Z*_0_(*q*) in the reciprocal space. In general, however, the transform *Z*_*j*_ is a complex, highly variable function of the vector **q**. In Fig. 2c and d, we have presented the real and imaginary parts of the function, respectively, for the vector **r**_**j**_ = (1, 1).

Substituting Eqs (5) and (6) into Eq. (4), we obtain the Fourier transform of pillar decorated surface in the form

7 $$Z({\bf{q}},{{\bf{r}}}_{{\bf{1}}},\ldots ,{{\bf{r}}}_{{\bf{N}}})=\frac{h}{2\pi }\frac{{J}_{1}(q)}{q}\sum _{j=1}^{N}\exp (\,-\,i{{\bf{r}}}_{{\bf{j}}}\cdot {\bf{q}}).$$

### PSD of pillar decorated surface

The PSD of a finite surface area equals^1^

8 $$C({\bf{q}})=\frac{{(2\pi )}^{2}}{A}\langle {|Z({\bf{q}})|}^{2}\rangle ,$$

where the angle brackets denote ensemble averaging. Substituting Eq. (7) into Eq. (8) and using the trigonometric form of complex numbers, we get

9 $$C({\bf{q}},N)=\frac{{h}^{2}}{A}{(\frac{{J}_{1}(q)}{q})}^{2}\langle {[\sum _{j=1}^{N}\cos ({{\bf{r}}}_{{\bf{j}}}\cdot {\bf{q}})]}^{2}+{[\sum _{j=1}^{N}\sin ({{\bf{r}}}_{{\bf{j}}}\cdot {\bf{q}})]}^{2}\rangle .$$

Please note that the available pillar positions are determined by the shape of the area *A*. Therefore, *C*(*q*, *N*) is dependent both on the area’s size and, implicitly, on its shape.

A simple transformation of Eq. (9) yields

10 $$C({\bf{q}},N)=\frac{N{h}^{2}}{A}{(\frac{{J}_{1}(q)}{q})}^{2}(1+T({\bf{q}},N)),$$

where

11 $$T({\bf{q}},N)=\langle \frac{1}{N}\sum _{l=1}^{N}\sum _{\begin{array}{c}m=1,\\ m\ne l\end{array}}^{N}\cos ({\bf{q}}\cdot {{\bf{r}}}_{{\bf{l}}{\bf{m}}})\rangle $$

and **r**_**lm**_ = **r**_**l**_ - **r**_**m**_. It is convenient to divide Eq. (10) by the factor *Nh*^2^/*A* and analyze the resulting function

12 $${C}^{\ast }({\bf{q}},N)={(\frac{{J}_{1}(q)}{q})}^{2}(1+T({\bf{q}},N)),$$

which is independent of the pillar height *h* and, as we will see later on, is relatively weakly dependent on the pillar number *N*.

As we can see, the function *T*(**q**, *N*) represents an ensemble averaged sum over all ordered pillar pairs. We can distinguish two subsets of the pairs. The first contains all the uncorrelated pillar pairs at a distance *r*_*lm*_ larger than some characteristic distance *r*_*c*_. This distance depends on the distribution of pillar positions on the surface. In systems of the structure consistent with the model of random sequential adsorption (RSA)^7^, e.g., the distance changes with surface coverage and, in practice, is always less than five^10^. The second subset contains all the other pillar pairs. Thus,

13 $$\langle \frac{1}{N}\sum _{l=1}^{N}\sum _{\begin{array}{c}m=1,\\ m\ne l\end{array}}^{N}\cos ({\bf{q}}\cdot {{\bf{r}}}_{{\bf{l}}{\bf{m}}})\rangle =\langle \frac{1}{N}\sum _{l=1}^{N}\sum _{\begin{array}{c}m=1,\\ m\ne l,\\ {r}_{lm}\le {r}_{c}\end{array}}^{N}\cos ({\bf{q}}\cdot {{\bf{r}}}_{{\bf{l}}{\bf{m}}})\rangle +\langle \frac{1}{N}\sum _{l=1}^{N}\sum _{\begin{array}{c}m=1,\\ m\ne l,\\ {r}_{lm} > {r}_{c}\end{array}}^{N}\cos ({\bf{q}}\cdot {{\bf{r}}}_{{\bf{l}}{\bf{m}}})\rangle .$$

**Figure 3:**
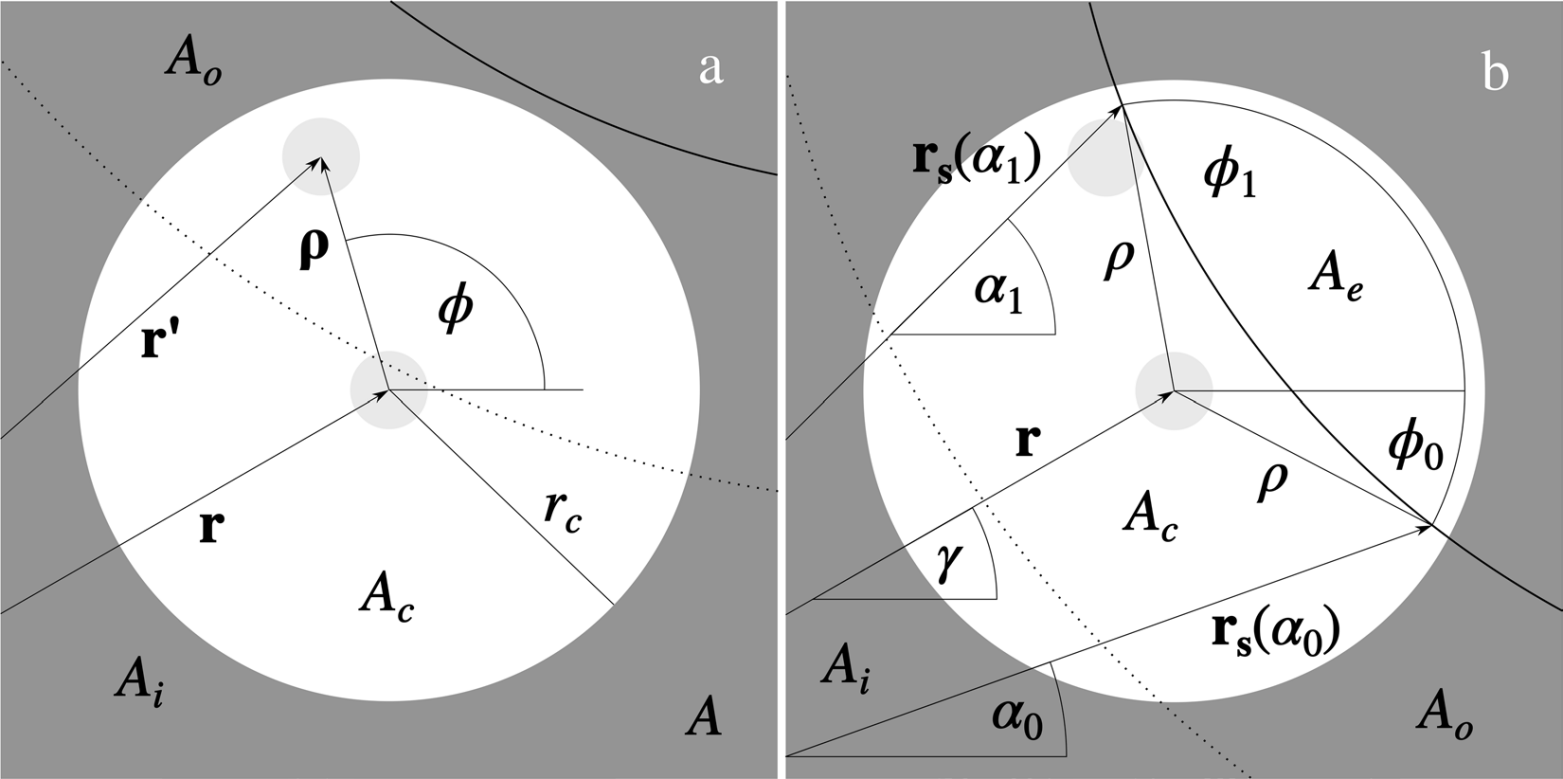
Geometry of two pillars at short separation distance (normal projection on the plane *z* = 0). **r** and **r**′ denote the pillars’ position vectors. *A*_*c*_ is a disk of the radius *r*_*c*_, centered at the point **r**. The thick, solid curves denote the boundary of the area *A*. The dotted lines denote the boundary between the inner and outer parts of the area *A*, *A*_*i*_ and *A*_*o*_, respectively. (**a**) The pillar at r is located in the inner area *A*_*i*_. (**b**) The pillar at r is located in the outer area *A*_*o*_. Please note that the area *A*_*e*_ extends outside the area *A*.

Let us first consider correlated pillar pairs. Again, we can distinguish two subsets of the pairs. The first contains all the ordered pairs with the first pillar located in the inner part *A*_*i*_ of the area *A*, separated from the boundary **r**_**s**_(*α*) by the distance *r*_*c*_ (see Fig. 3a). The contribution from these pillar pairs to the first term on the RHS of Eq. (13) equals

14 $${T}_{ci}({\bf{q}},N)=\frac{1}{N}{\int }_{{A}_{i}}n({\bf{r}}){\int }_{{A}_{c}}n({\bf{r}}^{\prime} ,{\bf{r}})\cos [{\bf{q}}\cdot ({\bf{r}}^{\prime} -{\bf{r}})]{\rm{d}}{\bf{r}}^{\prime} {\rm{d}}{\bf{r}},$$

where **r** and **r**′ are the position vectors of the first and second pillars located, respectively, in the area *A*_*i*_ and circular area *A*_*c*_ = *π r*_*c*_^2^ centered at the point **r**, *n*(**r**) is the ensemble averaged pillar areal number density at the point **r**, and *n*(**r**′, **r**) is the ensemble averaged pillar areal number density at the point **r**′ given a pillar at **r**.

Because of statistical homogeneity and local, at the scale of *r*_*c*_, statistical isotropy of the system, the pillar number densities are equal *n*(**r**) = *θ*/*π* and *n*(**r**′, **r**) = *g*(*ρ*, *θ*) *θ*/*π*, where *θ* = *π N*/*A* is the pillar surface coverage, *ρ* is the length of the vector **ρ** = **r**′ - **r**, and *g*(*ρ*, *θ*) is the radial distribution function. Please note that this function equals zero for *ρ* ≤ 2, therefore we can integrate over the entire area *A*_*c*_ without violating the condition of pillar non-overlapping. Thus, for any **r**, the pillar number density *n*(**r**′, **r**) is described by the same function of *ρ* and *θ* only. In this sense, the function is independent of **r**. The same is true for the argument of the cosine function appearing in Eq. (14). Therefore, we can separate the integration over *A*_*i*_ and *A*_*c*_. Considering this and using polar coordinates, we can rewrite Eq. (14) as

15 $${T}_{ci}({\bf{q}},\theta )=\frac{\theta {A}_{i}}{\pi A}{\int }_{0}^{{r}_{c}}\rho g(\rho ,\theta ){\int }_{0}^{2\pi }\cos [q\rho \,\cos (\beta -\varphi )]{\rm{d}}\varphi {\rm{d}}\rho ,$$

where *β* and *ϕ* are the phase angle of the vector **q** and polar angle of the vector **ρ**, respectively. We can express the inner integral in a closed-form^11^, Eq. 9.1.18;^12^, Eq. 10.9.1 to get

16 $${T}_{ci}(q,\theta )=2\theta \frac{{A}_{i}}{A}{\int }_{0}^{{r}_{c}}\rho g(\rho ,\theta ){J}_{0}(q\rho ){\rm{d}}\rho ,$$

where *J*_0_(*x*) is the zeroth order Bessel function of the first kind.

Next, let us consider the second subset of correlated, ordered pillar pairs with the first pillar located in the outer part *A*_*o*_ of the area *A*, close to the curve **r**_**s**_(*α*) (see Fig. 3b). Their contribution to the first term on the RHS of Eq. (13) equals

17 $${T}_{co}({\bf{q}},N)=\frac{1}{N}{\int }_{{A}_{o}}n({\bf{r}})\{{\int }_{{A}_{c}}n({\bf{r}}^{\prime} ,{\bf{r}})\cos [{\bf{q}}\cdot ({\bf{r}}^{\prime} -{\bf{r}})]{\rm{d}}{\bf{r}}^{\prime} -{\int }_{{A}_{e}({\bf{r}})}n({\bf{r}}^{\prime} ,{\bf{r}})\cos [{\bf{q}}\cdot ({\bf{r}}^{\prime} -{\bf{r}})]{\rm{d}}{\bf{r}}^{\prime} \}{\rm{d}}{\bf{r}},$$

where *A*_*e*_(**r**) denotes the external part of the disk *A*_*c*_, extending outside the area *A*. Using the same reasoning as for the area *A*_*i*_, we can express Eq. (17) as

18 $${T}_{co}({\bf{q}},\theta )=2\theta \frac{{A}_{o}}{A}{\int }_{0}^{{r}_{c}}\rho g(\rho ,\theta ){J}_{0}(q\rho ){\rm{d}}\rho -{T}_{e}({\bf{q}},\theta ),$$

where

19 $${T}_{e}({\bf{q}},\theta )=\frac{\theta }{\pi A}{\int }_{{A}_{o}}{\int }_{{\rho }_{0}({\bf{r}})}^{{r}_{c}}\rho g(\rho ,\theta ){\int }_{{\varphi }_{0}(\rho ,{\bf{r}})}^{{\varphi }_{1}(\rho ,{\bf{r}})}\cos [q\rho \,\cos (\beta -\varphi )]{\rm{d}}\varphi {\rm{d}}\rho {\rm{d}}{\bf{r}}.$$

Here, *ρ*_0_(**r**) denotes the minimum value of |**ρ**|, while *ϕ*_0_(*ρ*, **r**) and *ϕ*_1_(*ρ*, **r**) are the minimum and maximum values of the polar angle *ϕ* (see Fig. 3b):

20 $${\varphi }_{0}(\rho ,{\bf{r}})=\gamma -{\varepsilon }_{0}(\rho ,{\bf{r}})\,{\rm{a}}{\rm{n}}{\rm{d}}\,{\varphi }_{1}(\rho ,{\bf{r}})=\gamma +{\varepsilon }_{1}(\rho ,{\bf{r}}),$$

where *γ* is the polar angle of the vector **r** and, from the law of cosines,

21 $${\varepsilon }_{i}(\rho ,{\bf{r}})=\pi -\arccos \frac{{\rho }^{2}+{r}^{2}-{r}_{s}{({\alpha }_{i})}^{2}}{2\rho r},\,i=0,1.$$

The angles *α*_*i*_, appearing in Eq. (21), denote the minimum and maximum polar angles of the vector **r**_**s**_ for the given area *A*_*e*_ and value of *ρ*.

Summing up the contributions from both subsets of correlated pillar pairs, we get the first term on the RHS of Eq. (13) to be

22 $${T}_{c}({\bf{q}},\theta )=2\theta {\int }_{0}^{{r}_{c}}\rho g(\rho ,\theta ){J}_{0}(q\rho ){\rm{d}}\rho -{T}_{e}({\bf{q}},\theta ).$$

The second term on the RHS of Eq. (13) represents the contribution from uncorrelated pillar pairs. We can calculate it as a difference between the integral over all possible positions of the ordered pillar pairs and integral over the positions of correlated pairs at close distances:

$$\begin{array}{ccc}{T}_{u}({\bf{q}},N) & = & \frac{1}{N}{\int }_{A}n({\bf{r}}){\int }_{A}n({\bf{r}}^{\prime} )\cos [{\bf{q}}\cdot ({\bf{r}}^{\prime} -{\bf{r}})]{\rm{d}}{\bf{r}}^{\prime} {\rm{d}}{\bf{r}}\\  &  & -\,\frac{1}{N}{\int }_{A}n({\bf{r}}){\int }_{{A}_{c}}n({\bf{r}}^{\prime} )\cos [{\bf{q}}\cdot ({\bf{r}}^{\prime} -{\bf{r}})]{\rm{d}}{\bf{r}}^{\prime} {\rm{d}}{\bf{r}}\\  &  & +\,\frac{1}{N}{\int }_{{A}_{o}}n({\bf{r}}){\int }_{{A}_{e}({\bf{r}})}n({\bf{r}}^{\prime} )\cos [{\bf{q}}\cdot ({\bf{r}}^{\prime} -{\bf{r}})]{\rm{d}}{\bf{r}}^{\prime} {\rm{d}}{\bf{r}}\end{array}$$

or, in terms of surface coverage,

23 $${T}_{u}({\bf{q}},\theta )=\frac{\theta }{\pi A}{\int }_{A}{\int }_{A}g(\rho ,\theta )\cos ({\bf{q}}\cdot {\boldsymbol{\rho }}){\rm{d}}{\bf{r}}^{\prime} {\rm{d}}{\bf{r}}-2\theta {\int }_{0}^{{r}_{c}}\rho g(\rho ,\theta ){J}_{0}(q\rho ){\rm{d}}\rho +{T}_{e}({\bf{q}},\theta ).$$

Please note that Eq. (23) is correct whether or not we neglect the correlations between close positions of pillars, as long as we do so in *all* integrals. Therefore, for the sake of simplicity, we have neglected the correlations and assumed that everywhere in Eq. (23) *g*(*ρ*, *θ*) = 1, which is the correct value for uncorrelated pillar pairs. That yields

24 $$\begin{array}{ccc}{T}_{u}({\bf{q}},\theta ) & = & \frac{\theta }{\pi A}{\int }_{A}{\int }_{A}\cos ({\bf{q}}\cdot {\boldsymbol{\rho }}){\rm{d}}{\bf{r}}^{\prime} {\rm{d}}{\bf{r}}-2\theta {\int }_{0}^{{r}_{c}}\rho {J}_{0}(q\rho ){\rm{d}}\rho \\  &  & +\,\frac{\theta }{\pi A}{\int }_{{A}_{o}}{\int }_{{\rho }_{0}({\bf{r}})}^{{r}_{c}}\rho {\int }_{{\varphi }_{0}(\rho ,{\bf{r}})}^{{\varphi }_{1}(\rho ,{\bf{r}})}\cos [q\rho \,\cos (\beta -\varphi )]{\rm{d}}\varphi {\rm{d}}\rho {\rm{d}}{\bf{r}}.\end{array}$$

Summing up the contributions from correlated and uncorrelated pillar pairs, we get

25 $$T({\bf{q}},\theta )=\frac{\theta }{\pi A}{\int }_{A}{\int }_{A}\cos ({\bf{q}}\cdot {\boldsymbol{\rho }}){\rm{d}}{\bf{r}}^{\prime} {\rm{d}}{\bf{r}}+2\theta {\int }_{0}^{{r}_{c}}\rho h(\rho ,\theta ){J}_{0}(q\rho ){\rm{d}}\rho -\frac{\theta }{\pi A}{I}_{e}({\bf{q}},\theta ),$$

where

26 $$h(\rho ,\theta )=g(\rho ,\theta )-1$$

is the total correlation function^13^, p.72 and

27 $${I}_{e}({\bf{q}},\theta )={\int }_{{A}_{o}}{\int }_{{\rho }_{0}({\bf{r}})}^{{r}_{c}}\rho h(\rho ,\theta ){\int }_{{\varphi }_{0}(\rho ,{\bf{r}})}^{{\varphi }_{1}(\rho ,{\bf{r}})}\cos [q\rho \,\cos (\beta -\varphi )]{\rm{d}}\varphi {\rm{d}}\rho {\rm{d}}{\bf{r}}.$$

We can split the middle integral on the RHS of Eq. (25) into two pieces, the part over 0 ≤ *ρ* < 2 and the part over 2 ≤ *ρ* ≤ *r*_*c*_. In the former, *h*(*ρ*, *θ*) = −1 and therefore^11^, Eq. 9.1.30;^12^, Eq. 10.6.6

28 $${\int }_{0}^{{r}_{c}}\rho h(\rho ,\theta ){J}_{0}(q\rho ){\rm{d}}\rho =-\,2\frac{{J}_{1}(2q)}{q}+{I}_{c}(q,\theta ),$$

where

29 $${I}_{c}(q,\theta )={\int }_{2}^{{r}_{c}}\rho h(\rho ,\theta ){J}_{0}(q\rho ){\rm{d}}\rho .$$

With that, we can rewrite Eq. (25) in the form

30 $$T({\bf{q}},\theta )=\frac{\theta }{\pi A}{\int }_{A}{\int }_{A}\cos ({\bf{q}}\cdot {\boldsymbol{\rho }}){\rm{d}}{\bf{r}}^{\prime} {\rm{d}}{\bf{r}}-4\theta \frac{{J}_{1}(2q)}{q}+2\theta {I}_{c}(q,\theta )-\frac{\theta }{\pi A}{I}_{e}({\bf{q}},\theta ).$$

Substituting Eq. (30) into Eq. (12) we get the general equation for the reduced PSD

31 $${C}^{\ast }({\bf{q}},\theta )={(\frac{{J}_{1}(q)}{q})}^{2}[1+\frac{\theta }{\pi A}{\int }_{A}{\int }_{A}\cos ({\bf{q}}\cdot {\boldsymbol{\rho }}){\rm{d}}{\bf{r}}^{\prime} {\rm{d}}{\bf{r}}-4\theta \frac{{J}_{1}(2q)}{q}+2\theta {I}_{c}(q,\theta )-\frac{\theta }{\pi A}{I}_{e}({\bf{q}},\theta )].$$

Let us comment on the physical meaning of the five terms of the sum appearing on the RHS of Eq. (31). The first, constant term represents the contribution from single pillars. The second term represents the contribution from all ordered pillar pairs with neglected overlapping and correlations between pillar positions. Thus, in this integral we have taken into account also the pairs of overlapping pillars and we have neglected deviations of the pillar number density at the distance *ρ* > 2. The third component is a correction term for pillar overlapping. The fourth component is a correction term for correlations between pillar positions. As we can see in Eq. (28), we have calculated the two correction terms by the integration over the radius *r*_*c*_ of the disk *A*_*c*_. As a consequence, we have integrated over the second pillar position extending outside the total integration area *A*, which leads to an overestimation of the two terms. To compensate the overestimation, we have subtracted the fifth term, which is the integral over the outer area *A*_*o*_ and external part of the disk, *A*_*e*_. Thus, the fifth component is a correction term for the third and fourth components.

If, for any *α*, *r*_*s*_(*α*) ≫ *r*_*c*_, then *A*_*o*_ ≪ *A* and the last term of the sum on the RHS of Eq. (31) becomes negligibly small. Then, in the case of large surface area, Eq. (31) simplifies to

32 $${C}^{\ast }({\bf{q}},\theta )={(\frac{{J}_{1}(q)}{q})}^{2}[1+\frac{\theta }{\pi A}{\int }_{A}{\int }_{A}\cos ({\bf{q}}\cdot {\boldsymbol{\rho }}){\rm{d}}{\bf{r}}^{\prime} {\rm{d}}{\bf{r}}-4\theta \frac{{J}_{1}(2q)}{q}+2\theta {I}_{c}(q,\theta )].$$

If, on the other hand, *θ* ≪1, the total correlation function at *ρ* > 2 tends to zero. Then, in the case of low surface coverage, the integral *I*_*c*_(*q*, *θ*) → 0 and we get

33 $${C}^{\ast }({\bf{q}},\theta )={(\frac{{J}_{1}(q)}{q})}^{2}[1+\frac{\theta }{\pi A}{\int }_{A}{\int }_{A}\cos ({\bf{q}}\cdot {\boldsymbol{\rho }}){\rm{d}}{\bf{r}}^{\prime} {\rm{d}}{\bf{r}}-4\theta \frac{{J}_{1}(2q)}{q}-\frac{\theta }{\pi A}{I}_{e0}({\bf{q}})],$$

where

34 $${I}_{e0}({\bf{q}})=-{\int }_{{A}_{o0}}{\int }_{{\rho }_{0}({\bf{r}})}^{2}\rho {\int }_{{\varphi }_{0}(\rho ,{\bf{r}})}^{{\varphi }_{1}(\rho ,{\bf{r}})}\cos [q\rho \,\cos (\beta -\varphi )]{\rm{d}}\varphi {\rm{d}}\rho {\rm{d}}{\bf{r}}$$

is the correction term for excess pillar overlapping in the term 4*θ J*_1_(2*q*)/*q*. Here, *A*_*o*__0_ denotes the outer part of *A*_*o*_, extending over a distance less than two from **r**_**s**_(*α*).

If *A*_*o*_ ≪ *A* and *θ* ≪ 1, Eq. (31) simplifies to

35 $${C}^{\ast }({\bf{q}},\theta )={(\frac{{J}_{1}(q)}{q})}^{2}[1+\frac{\theta }{\pi A}{\int }_{A}{\int }_{A}\cos ({\bf{q}}\cdot {\boldsymbol{\rho }}){\rm{d}}{\bf{r}}^{\prime} {\rm{d}}{\bf{r}}-4\theta \frac{{J}_{1}(2q)}{q}].$$

Please note that the equations derived in this section are valid also for randomly distributed cylindrical disks and cavities.

### PSD of pillar decorated circular area

Equations (31)–(35) are valid for surfaces of various shape. In what follows, we have restricted ourselves to a disk of a radius *r*_*s*_, centered at the origin. Then, we can derive a closed-form expression for the second term of the sum on the RHS of Eq. (31), representing the contribution from all pillar pairs as an explicit function of *r*_*s*_:

36 $$\begin{array}{ccc}{T}_{a}(q,\theta ,{r}_{s}) & = & \frac{\theta }{{\pi }^{2}{r}_{s}^{2}}{\int }_{0}^{{r}_{s}}{\int }_{0}^{2\pi }{\int }_{0}^{{r}_{s}}{\int }_{0}^{2\pi }\cos \,[q\,\cos (\beta )(r^{\prime} \,\cos (\gamma ^{\prime} )-r\,\cos (\gamma ))\\  &  & +\,q\,\sin (\beta )(r^{\prime} \,\sin (\gamma ^{\prime} )-r\,\sin (\gamma ))]{\rm{d}}\gamma ^{\prime} {\rm{d}}r^{\prime} {\rm{d}}\gamma {\rm{d}}r.\end{array}$$

We can transform the integrand to obtain

37 $$\begin{array}{ccc}{T}_{a}(q,\theta ,{r}_{s}) & = & \frac{\theta }{{\pi }^{2}{r}_{s}^{2}}({\{{\int }_{0}^{{r}_{s}}{\int }_{0}^{2\pi }\cos [qr\cos (\beta -\gamma )]{\rm{d}}\gamma {\rm{d}}r\}}^{2}\\  &  & +\,{\{{\int }_{0}^{{r}_{s}}{\int }_{0}^{2\pi }\sin [qr\cos (\beta -\gamma )]{\rm{d}}\gamma {\rm{d}}r\}}^{2}).\end{array}$$

Integration over *γ* leads to zeroing of the second integral^11^, Eq. 9.1.45;^12^, Eq. 10.12.3 and gives^11^, Eq. 9.1.18;^12^, Eq. 10.9.1

38 $${T}_{a}(q,\theta ,{r}_{s})=\frac{4\theta }{{r}_{s}^{2}}{[{\int }_{0}^{{r}_{s}}r{J}_{0}(qr){\rm{d}}r]}^{2}.$$

Integrating over *r*, we finally obtain^11^, Eq. 9.1.30;^12^, Eq. 10.6.6

39 $${T}_{a}(q,\theta ,{r}_{s})=4\theta {(\frac{{J}_{1}(q{r}_{s})}{q})}^{2}.$$

Moreover, because of the circular symmetry of the area *A*, the functions *ε*_0_(*ρ*, **r**) and *ε*_1_(*ρ*, **r**) simplify to

40 $$\varepsilon (\rho ,r,{r}_{s})=\pi -\arccos \frac{{\rho }^{2}+{r}^{2}-{r}_{s}^{2}}{2\rho r},$$

which leads to the following expressions for the limiting values of the angle *ϕ*:

41 $${\varphi }_{0}(\rho ,r,{r}_{s},\gamma )=\gamma -\varepsilon (\rho ,r,{r}_{s})\,{\rm{a}}{\rm{n}}{\rm{d}}\,{\varphi }_{1}(\rho ,r,{r}_{s},\gamma )=\gamma +\varepsilon (\rho ,r,{r}_{s}).$$

With that, we can write the integral *I*_*e*_ in the form

42 $${I}_{e}({\bf{q}},\theta ,{r}_{s})={\int }_{{r}_{s}-{r}_{c}}^{{r}_{s}}r{\int }_{{r}_{s}-r}^{{r}_{c}}\rho h(\rho ,\theta ){\int }_{0}^{2\pi }{\int }_{\gamma -\varepsilon (\rho ,r,{r}_{s})}^{\gamma +\varepsilon (\rho ,r,{r}_{s})}\cos [q\rho \,\cos (\beta -\varphi )]{\rm{d}}\varphi {\rm{d}}\gamma {\rm{d}}\rho {\rm{d}}r.$$

**Figure 4:**
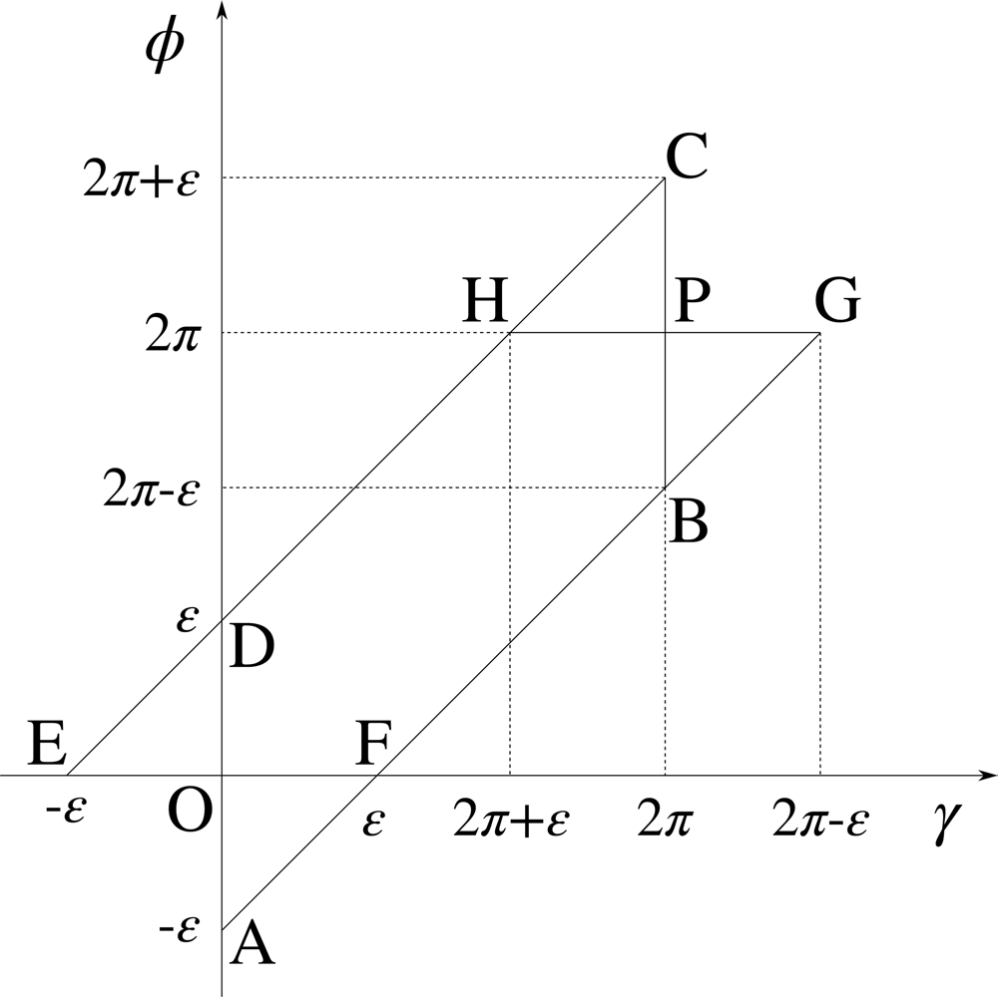
Domains of integration over the angles *γ* and *ϕ*. Because of the property cos[*z* cos(*x* + *π*)] = cos[*z* cos(*x*)], integration over the triangles AFO and CHP is equivalent to integration over the triangles BGP and DEO, respectively. Consequently, integration over the area ABCD is equivalent to integration over the area EFGH.

As shown in Fig. 4, we can exploit the property cos[*z* cos(*x* + *π*)] = cos[*z* cos(*x*)], where *x* and *z* are arbitrary real numbers, to express the innermost, double integral as

43 $${\int }_{0}^{2\pi }{\int }_{\gamma -\varepsilon (\rho ,r,{r}_{s})}^{\gamma +\varepsilon (\rho ,r,{r}_{s})}\cos [q\rho \,\cos (\beta -\varphi )]{\rm{d}}\varphi {\rm{d}}\gamma ={\int }_{0}^{2\pi }{\int }_{\varphi -\varepsilon (\rho ,r,{r}_{s})}^{\varphi +\varepsilon (\rho ,r,{r}_{s})}\cos [q\rho \,\cos (\beta -\varphi )]{\rm{d}}\gamma {\rm{d}}\varphi .$$

We can solve the latter in a closed-form^11^, Eq. 9.1.18;^12^, Eq. 10.9.1 to finally get

44 $${I}_{e}(q,\theta ,{r}_{s})=4\pi {\int }_{{r}_{s}-{r}_{c}}^{{r}_{s}}r{\int }_{{r}_{s}-r}^{{r}_{c}}\rho h(\rho ,\theta )\varepsilon (\rho ,r,{r}_{s}){J}_{0}(q\rho ){\rm{d}}\rho {\rm{d}}r.$$

Substituting Eq. (39) into Eq. (31), we get the following equation for the reduced PSD of circular area:

45 $${C}^{\ast }(q,\theta ,{r}_{s})={(\frac{{J}_{1}(q)}{q})}^{2}[1+4\theta {(\frac{{J}_{1}(q{r}_{s})}{q})}^{2}-4\theta \frac{{J}_{1}(2q)}{q}+2\theta {I}_{c}(q,\theta )-\frac{\theta }{{\pi }^{2}{r}_{s}^{2}}{I}_{e}(q,\theta ,{r}_{s})],$$

where the integrals *I*_*c*_(*q*, *θ*) and *I*_*e*_(*q*, *θ*, *r*_*s*_) are given by Eqs (29) and (44), respectively. Please note that this function is independent of wave-vector direction, as we have expected for the system of circular symmetry.

Thus, in general, to get the reduced PSD, we need to calculate the integrals *I*_*c*_(*q*, *θ*) and *I*_*e*_(*q*, *θ*, *r*_*s*_). As we can see from Eqs (29) and (44), because of the complicated integrands dependent on the correlation function of an unknown analytical form, we can compute them numerically only.

The limiting forms of Eq. (45) for *A*_*o*_ ≪ *A* and/or *θ* ≪ 1, corresponding to the general Eqs (32)–(35), become:

46 $${C}^{\ast }(q,\theta ,{r}_{s})={(\frac{{J}_{1}(q)}{q})}^{2}[1+4\theta {(\frac{{J}_{1}(q{r}_{s})}{q})}^{2}-4\theta \frac{{J}_{1}(2q)}{q}+2\theta {I}_{c}(q,\theta )],$$

47 $${C}^{\ast }(q,\theta ,{r}_{s})={(\frac{{J}_{1}(q)}{q})}^{2}[1+4\theta {(\frac{{J}_{1}(q{r}_{s})}{q})}^{2}-4\theta \frac{{J}_{1}(2q)}{q}-\frac{\theta }{{\pi }^{2}{r}_{s}^{2}}{I}_{e0}(q,{r}_{s})],$$

48 $${I}_{e0}(q,{r}_{s})=-\,4\pi {\int }_{{r}_{s}-2}^{{r}_{s}}r{\int }_{{r}_{s}-r}^{2}\rho \varepsilon (\rho ,r,{r}_{s}){J}_{0}(q\rho ){\rm{d}}\rho {\rm{d}}r,$$

and

49 $${C}^{\ast }(q,\theta ,{r}_{s})={(\frac{{J}_{1}(q)}{q})}^{2}[1+4\theta {(\frac{{J}_{1}(q{r}_{s})}{q})}^{2}-4\theta \frac{{J}_{1}(2q)}{q}],$$

respectively.

## Numerical Verification

In principle, we can use Eq. (45) to determine important parameters of pillar layers. Specifically, we can find the pillar radius and height, disk radius, and surface coverage. For that, we have first to determine the function of height of the pillar layer of interest by, e.g., scanning its surface. Then, we need to numerically calculate the discrete Fourier transform of the function of height. There are a few open-source packages available for this purpose. Perhaps the most common is the package FFTW^14,15^. In our earlier paper, we described the application of this package in calculating the PSD of spherical particle monolayer^16^. Input data to the programs should usually be in the form of a rectangular array of *z*-coordinates of the scanned rectangular area *A*_*s*_. Therefore, to ensure a circular shape of the pillar decorated surface, we need to assign the height *z* = 0 to all points located outside the analyzed disk area *A*. Finally, we have to fit the dimensional version of Eq. (45), i.e.,

50 $$\begin{array}{ccc}S(Q,\theta ,{R}_{s},a,H) & = & \frac{\theta {R}_{s}^{2}}{{A}_{s}}{(\frac{{J}_{1}(Qa)H}{Q})}^{2}[1+4\theta {(\frac{{J}_{1}(Q{R}_{s})}{Qa})}^{2}-4\theta \frac{{J}_{1}(2Qa)}{Qa}\\  &  & +\,2\theta {I}_{c}(Qa,\theta )-\theta {(\frac{a}{\pi {R}_{s}})}^{2}{I}_{e}(Qa,\theta ,{R}_{s}/a)]\end{array}$$

to the numerical results of discrete Fourier transform. In Eq. (50), *Q* = *q*/*a*, *H* = *ha*, *R*_*s*_ = *r*_*s*_*a*, and *S* = *Ca*^4^ are the dimensional wave number, pillar height, disk radius, and PSD, respectively. Please note that, to take into account the non-circular shape of the scanned area, we have to replace the factor *θ*/*π*, appearing in the dimensional equation for the PSD, with the factor *θR*_*s*_^2^/*A*_*s*_.

In general, the application of Eq. (50) requires explicit analytical expressions for the integrals *I*_*c*_(*q*, *θ*) and *I*_*e*_(*q*, *θ*, *r*_*s*_) that are 2D and 3D, highly variable functions of wavenumber. To find the expressions for a specific radial distribution function, we have to numerically calculate the integrals for a number of *θ* and *r*_*s*_ values, over a range of wavenumber. Then, we can use the least-square method to fit analytical expressions to the numerical results. This procedure is out of the scope of this paper. We are going to present numerical results of such calculations for pillar layers consistent with the RSA distribution in a future report. Our preliminary results suggest, however, that the simplified form of Eq. (50)

51 $$S(Q,\theta ,{R}_{s},a,H)=\frac{\theta {R}_{s}^{2}}{{A}_{s}}{(\frac{{J}_{1}(Qa)H}{Q})}^{2}[1+4\theta {(\frac{{J}_{1}(Q{R}_{s})}{Qa})}^{2}-4\theta \frac{{J}_{1}(2Qa)}{Qa}],$$

with the integrals *I*_*c*_(*q*, *θ*) and *I*_*e*_(*q*, *θ*, *r*_*s*_) neglected, is sufficient to determine the parameters *a* and *R*_*s*_ with a relative standard error less than 1%, at any surface coverage and for any size of analyzed surface as long as *r*_*s*_ ≥ *r*_*c*_. To achieve this accuracy, the pillar number *N* must be large enough and therefore, at low surface coverage, ensemble averaging can be necessary.

**Figure 5:**
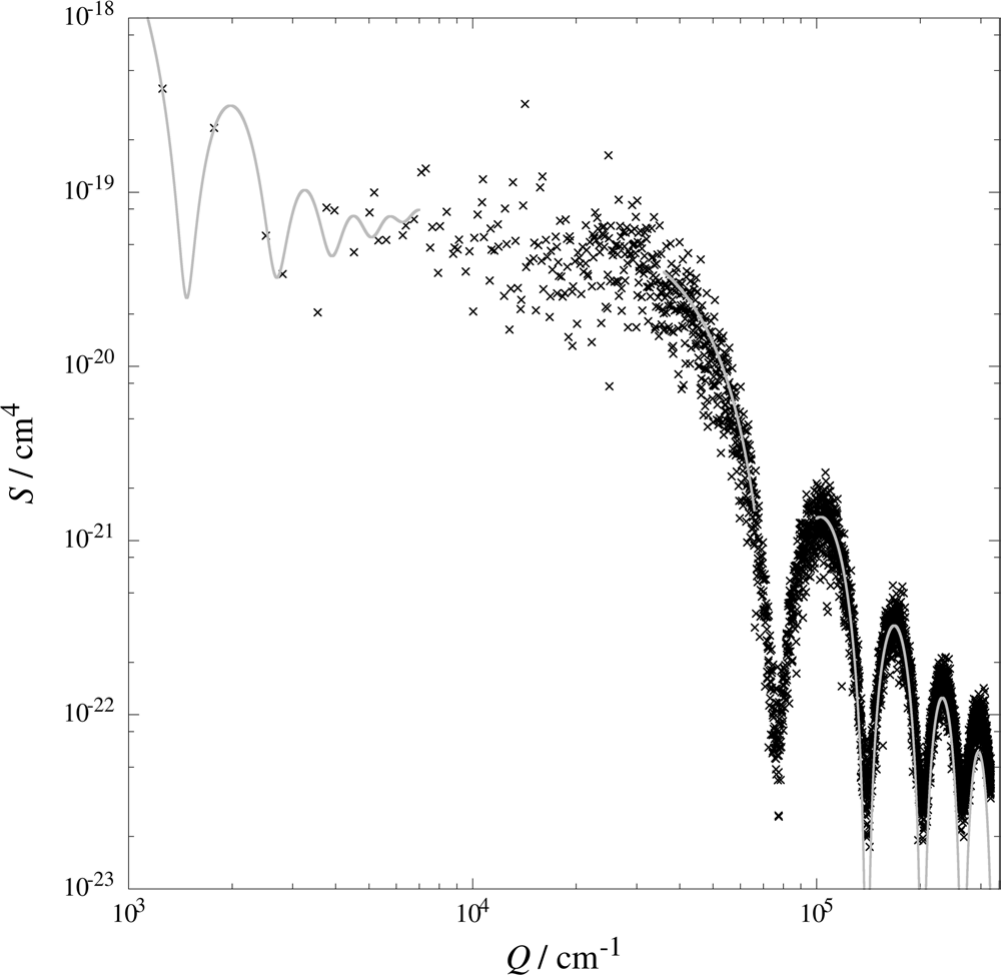
PSD of a circular area decorated with randomly distributed pillars. Points denote numerical results derived, through discrete Fourier transform of 512^2^ height measurements, from a virtual pillar system with the parameters *a* = 0.5 μm, *H* = 1 μm, *A*_*s*_ = 2500 μm^2^, *R*_*s*_ = 25 μm, and *θ* = 0.05. Lines represent least-square fits to the numerical data in the low-, mid-, and high-wavenumber ranges^20^. In the lower and upper ranges, for *Q* < 7000 cm^−1^ and *Q* > 10^5^ cm^−1^, respectively, we have fitted Eq. (51). In the mid-range, for 3.6 × 10^4^ cm^−1^ < *Q* < 6.6 × 10^4^ cm^−1^, we have used Eq. (52).

Figure 5 presents a comparison of PSD calculated numerically with the library FFTW (points) and fits of Eq. (51) to the numerical data (lines). We have derived the numerical results from a virtual system of 160 pillars of the radius *a* = 0.5 μm and height *H* = 1 μm, randomly distributed over a square area *A*_*s*_ = 2500 μm^2^, created according to the RSA model. Thus, the pillar surface coverage in this system is low and equals *θ* = 0.05. We have determined the height of the system at 512^2^ grid points equidistantly distributed over the area *A*_*s*_. To calculate the PSD for a circular area, we have set *z* = 0 at all grid points outside the disk of the radius *R*_*s*_ = 25 μm, inscribed in the area *A*_*s*_. At this value of *R*_*s*_, corresponding to *r*_*s*_ = 50, neglecting the integral *I*_*e*_(*q*, *θ*, *r*_*s*_) results in the PSD relative error less than 1%, as we have found in our preliminary calculations. Then, using FFTW library, we have calculated discrete Fourier transform of the data. Finally, we have calculated powers of all wave-vectors and summed them over narrow wavenumber intervals to calculate the discrete values of PSD. To back determine the system parameters, we have fitted Eq. (51) to the numerical data over low- and high-wavenumber ranges where the PSD shows numerous, sharp minima determined by the Bessel functions appearing in Eqs. (50) and (51). From the general properties of the function *J*_1_(*x*) we can deduce that in the limit of small wavenumber, where *Qa* ≪ 1, the PSD is determined by the term (*J*_1_(*QR*_*s*_)/*Qa*)^2^ with positions of minima dependent on *R*_*s*_. Thus, fitting the data in the range of low-wavenumber, we can find the disk radius. In the limit of large wavenumer, on the other hand, the PSD is determined by the factor (*J*_1_(*Qa*)/*Q*)^2^ with zeros dependent on *a*. Therefore, fitting the data in the high-wavenumber range, we can find the pillar radius. In our system, we have obtained the following radii and standard errors: *a* = 0.5007 ± 0.0007 μm and *R*_*s*_ = 25.93 ± 0.21 μm. The fitting results are quite consistent with the original values used in our RSA simulation.

The PSD simplicity in the limiting wavenumber ranges has also a negative consequence: the parameters *H* and *θ* are strongly correlated and their standard errors are usually very large. Moreover, sampling of the PSD in the low-wavenumber range is poor. In the high-wavenumber range, on the other hand, the calculated PSD of the rapidly changing function of height can be aliased because the discrete Fourier transform of such function is not bandwidth limited^17^, p. 494. Therefore, to determine the pillar height and surface coverage, we have to carry out the fitting procedure over a mid-wavenumber range. In our system, we have chosen the wavenumber interval 3.6 × 10^4^ cm^−1^ < *Q* < 6.6 × 10^4^ cm^−1^. We have fixed the radii at *a* = 0.5007 μm and *R*_*s*_ = 25.93 μm, according to the results of fitting in the lower and upper ranges. Also, to eliminate the PSD suppression with the increase in *Q*, we have fitted the reduced form of Eq. (51), i.e.,

52 $${S}^{\ast }(Q,\theta ,{R}_{s},a,H)=\frac{\theta {R}_{s}^{2}{H}^{2}}{{A}_{s}}[1+4\theta {(\frac{{J}_{1}(Q{R}_{s})}{Qa})}^{2}-4\theta \frac{{J}_{1}(2Qa)}{Qa}],$$

to the numerical data divided by the factor (*J*_1_(*Qa*)/*Q*)^2^. The results of fitting, *H* = 1 ± 1.3 μm and *θ* = 0.05 ± 0.13, are in agreement with the original values used in our simulation. The large standard errors result from a large scatter of the numerical results, evident in Fig. 5, which is a consequence of the small pillar number in our system.

## Conclusion

Our results suggest that the PSD can be a valuable source of quantitative information on parameters of planar surfaces decorated with randomly distributed, cylindrical pillars, disks, or cavities. In the case of statistically isotropic, circular surface area, the PSD provides us with the radius of the area, dimensions of the pillar, and surface coverage. The analysis presented here provides means to design and produce surfaces of controlled roughness. To better exploit the results, we need to numerically calculate the integrals appearing in the equation for the PSD and to approximate them with analytical, 2D and 3D functions of the wavenumber, pillar surface coverage, and disk radius. That will be a subject of our future investigation.

## Data Availability

All data generated and analysed during the current study are available from the corresponding author on reasonable request.
